# Mayer-Rokitansky-Kuster-Hauser syndrome

**DOI:** 10.61622/rbgo/2025FPS4

**Published:** 2025-05-16

**Authors:** Claudia Cristina Takano Novoa, Mila Torii Correa Leite, Marair Gracio Ferreira Sartori

**Affiliations:** Escola Paulista de Medicina Universidade Federal de São Paulo São Paulo SP Brazil Escola Paulista de Medicina, Universidade Federal de São Paulo, São Paulo, SP, Brazil; Escola Paulista de Medicina Universidade Federal de São Paulo São Paulo SP Brazil Escola Paulista de Medicina, Universidade Federal de São Paulo, São Paulo, SP, Brazil; Escola Paulista de Medicina Universidade Federal de São Paulo São Paulo SP Brazil Escola Paulista de Medicina, Universidade Federal de São Paulo, São Paulo, SP, Brazil

## Abstract

•Mayer-Rokitansky-Kuster-Hauser syndrome (MRKH) is the leading cause of vaginal agenesis.

•It is characterized by primary amenorrhea with typical adrenarche and telarche and may be associated with congenital urological and skeletal conditions that should be investigated.

•Differential diagnoses include: vaginal obstructions (imperforate hymen, distal vaginal atresia, transverse vaginal septum), uterine obstructions (cervical atresia), and differences in sexual development (gonadal dysgenesis, complete androgen insensitivity and congenital adrenal hyperplasia due to CYP17 deficiency).

•Laboratory tests (testosterone, follicle-stimulating hormone [FSH] and karyotype) and radiological tests (pelvic ultrasound and MRI) are necessary.

•Vaginal dilation is the first line of treatment with high success rates.

**Recommendations**

•The initial course of action consists of proper guidance on the syndrome, treatment options and clarification on sexual relationships and reproductive future.
•Treatment can be offered in late adolescence or early adulthood, considering the maturity to adhere to the procedures and the desire to create a neovagina.
•The first line of treatment should be vaginal dilation. Multidisciplinary care is preferred and supervision of treatment with dilators in conjunction with physiotherapy is always beneficial.
•Surgical creation of a neovagina should be reserved for patients who have not been successful with dilation or for those who prefer surgery after proper guidance and a shared decision with the medical team. When surgical treatment is indicated, the patient should be referred to referral centers.
•All patients should have access to psychological support and be encouraged to seek support groups.

## Background

Mayer-Rokitansky-Kuster-Hauser syndrome (MRKH) is the leading cause of vaginal agenesis, affecting one in every 5,000 women.^(1)^ In this congenital condition, genetic alterations affect the development of the Müllerian ducts during the embryonic period, resulting in the complete absence or significant hypoplasia of the uterus and vagina, with normal development of the external genitalia and breasts. The vaginal canal is short or there is only a small introitus below the urethra. There may be a remnant uterus or uterine horns with or without endometrium. The ovaries have adequate structure and function. The diagnosis is made by investigating primary amenorrhea with typical adrenarche and thelarche. Carriers have adequate height, normal breast development, and typical distribution of hairiness and external genitalia. On physical examination, the vagina is short with no palpable cervix or only a small concavity (“dimple”). Differential diagnoses include: vaginal obstructions (imperforate hymen, distal vaginal atresia, transverse vaginal septum), uterine obstructions (cervical atresia), and differences in sexual development (complete androgen insensitivity and CYP17A1 deficiency).^(2)^

### What is the gynecologic physical assessment like?

In MRKH, there is typical hairiness and genitalia, adequate breast development, and the presence of a short vagina or small blind concavity (“dimple”).In imperforate hymen, the hymenal membrane is protruding and bluish. In obstructive vaginal and uterine conditions, there are symptoms of cyclical and progressive abdominal or pelvic pain and palpation of a pelvic mass is frequent, due to the presence of hematometra. The physical examination can be similar, with the vaginal canal absent or of varying lengths, with a blind fundus, without identification of the cervix either in the transverse septa, or in distal vaginal atresia and cervical atresia. Radiological examinations (ultrasound and magnetic resonance imaging of the pelvis) are necessary to help make the differential diagnosis between vaginal and uterine obstructions, since the physical examination can be similar.^(2,3)^

Complete androgen insensitivity also presents as primary amenorrhea, short vagina and absence of cervix, adequate breast development, but with little axillary and pubic hair. Although the testicular gonads are usually intra-abdominal, they can be palpated in the inguinal canal, accompanied by inguinal hernias.

Complete gonadal dysgenesis presents as primary amenorrhea, with inadequate breast development for age and little axillary and pubic hair growth. The gonads are dysgenic (ribbon ovary) and gonadectomy is recommended after puberty to avoid malignancy. The karyotype may be 46,XY or 45 XO, XY.

17-alpha-hydroxylase deficiency (CYP17A1) is a very rare form of congenital adrenal hyperplasia, characterized by glucocorticoid deficiency, mineralocorticoid excess and sex steroid deficiency (hypergonadotrophic hypogonadism). Primary amenorrhoea occurs with failure in pubertal development.

### How to perform the clinical investigation?

The following tests should be requested:

Laboratory tests:

- Testosterone;
- FSH;
- Karyotype.

Radiological tests:

- Pelvic and urinary tract ultrasound;
- Pelvic magnetic resonance imaging (MRI).

### Are there other congenital conditions associated with MRKH?

Among patients with MRKH, up to 53% have other associated congenital conditions, mainly urological (27%-29%) and skeletal (8%-32%).^(4)^ There is an incidence, although less frequent, of hearing loss, which is also found in VATER/VACTERL syndrome (vertebral defects, anal atresia, cardiac defects, tracheoesophageal fistula, renal anomalies and limb abnormalities).^(5)^

Ultrasound of the kidneys and urinary tract and radiography of the spine should be requested.

### Can rudimentary Müllerian structures be found in MRKH?

Rudimentary Müllerian structures can be found on MRI in 90% of patients, but may not be identified by ultrasound, especially before puberty.^(6)^ Magnetic resonance imaging can be requested without contrast and analyzed by an experienced radiologist.

### Should diagnostic laparoscopy be indicated?

Diagnostic laparoscopy is not necessary in MRKH. It may be indicated for cases of endometriosis due to retrograde menstruation of obstructed uterine horns. Although hormonal suppression improves cyclic pain and endometriosis in most patients, in the absence of cervix and upper third of the vagina (unilateral or bilateral), removal of obstructed uterine bodies with active endometrium may be necessary.^(7)^

### Is psychological support necessary?

Psychological support should be offered to all patients, as well as encouragement to seek collective and/or individual support groups. Many patients experience anxiety and depression, question their identity and have difficulty in dealing with infertility.

### How to provide guidance on reproductive aspects?

Issues such as adoption and surrogate uterus should be addressed as future options for having children.^(8)^ Uterine transplantation, although still performed on an experimental basis, is currently a viable treatment, but it is associated with surgical and immunosuppression-related risks that must be considered.^(9)^

### What are the treatment options for MRKH?

The first line of treatment should be vaginal dilation with an expected success rate of 90%-96%.^(3)^ When well supervised, almost all patients can achieve adequate vaginal length quickly. Surgical creation of a neovagina should be reserved for patients who have not had success with dilation or those who prefer surgery after discussion with the medical team.^(3-11)^

### When should a neovagina be performed?

Treatment can be offered in late adolescence or early adulthood. Considering that even in surgical treatment, vaginal dilation is necessary in the postoperative period, treatment should be initiated when there is sufficient maturity for consent and adherence to the procedure.^(3)^

### How to guide vaginal dilation?

**Figure 1:**
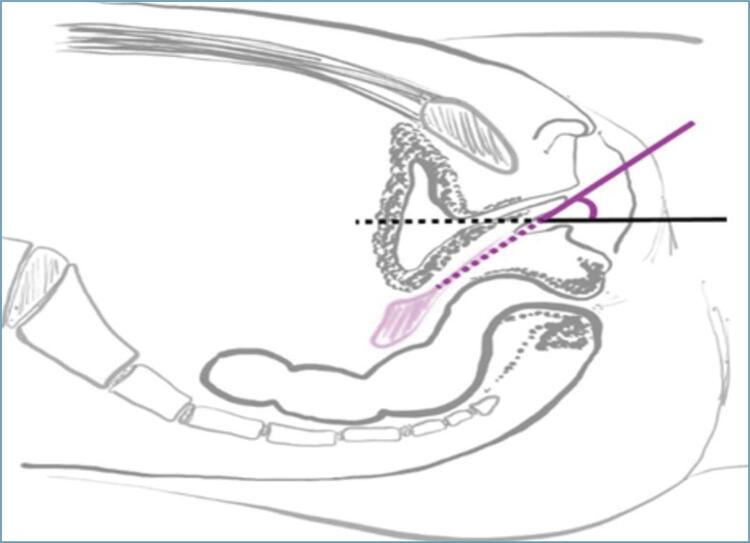
Positioning of the dilator in the distal vagina. Note the correct direction of the dilator on the pink line at an angle to the horizontal plane (black line).

Source: Marta Maria Kemp^(^^12^^)^

According to the dilation protocol for vaginal agenesis suggested by the American College of Obstetricians and Gynecologists – ACOG (2018),^(3)^ the patient should be instructed by the physician with the aid of a mirror to be able to identify the clitoris, urethra and distal vagina. Then, she is instructed to place the dilator in the distal vagina at an appropriate angle and press it until feeling discomfort, without compressing the urethra or rectum (Figure 1).

The patient should maintain the pressure intermittently and progressively for approximately 20 minutes once a day, and privacy strategies should be discussed.^(3)^

Medical follow-up should be performed every one to two weeks during the first month, and then monthly until the adequate vaginal length is reached (between 6 and 8 cm). There is no minimum vaginal length to initiate sexual activity. Follow-up with a pelvic physiotherapist is always beneficial, but it becomes essential when there is associated genito-pelvic pain.^(3,11,12)^

### How long does it take until reaching an adequate length of the neovagina with the use of dilators?

The time needed until reaching an adequate length of the vaginal canal depends on adherence to the dilation procedure and can vary between two months and two years. Therefore, it is extremely important that each patient decides the best time to begin the treatment. Failure to dilate occurs when there is no understanding of the technique and/or when there is no appropriate supervision by a multidisciplinary team.^(3,10)^ The use of molds made by 3D printer in an attempt to individualize cases is a recent option.^(12)^

### How are surgical neovaginas performed? What are the surgical complications?

Surgeries to create neovaginas should be reserved for rare cases in which vaginal dilation has not been successful or for patients who have jointly decided on the procedure with the multidisciplinary team. The use of vaginal molds will be necessary after the surgical procedure, so the information that surgical success will depend on this procedure must be properly clarified.

There are several surgical techniques for neovaginas, and the choice will depend on the experience of each professional.

One of the most commonly performed surgeries is the modified Abbe-McIndoe technique, which consists of dissecting a space between the rectum and the bladder, which is then covered with a skin graft.^(13)^ It has the advantages of shorter surgical time and lower morbidity, compared to the surgical abdominal access. Currently, a vaginal mold covered with oxidized cellulose has been used to cover the open area with excellent results, without the harmful effects of using skin grafts, such as scarring in the donor area, fibrosis, retraction and necrosis.^(14,15)^ Oxidized cellulose allows adequate epithelialization without adhesion between the mold and the dissected area. Six months after surgery, the neovagina is histologically identical to a normal vagina.^(12,14)^

Other techniques involve the use of devices placed laparoscopically to promote vaginal elongation by traction (Vecchietti procedure),^(16)^ the use of peritoneum to cover the neovagina wall (Davydov procedure)^(17)^ and neovaginoplasties with intestinal segments.^(18)^ There are descriptions of other lining materials such as amnion,^(19)^ natural latex^(20)^ and tilapia skin^(21)^ in a case series.

Each surgical technique chosen presents morbidity associated with the complexity of the procedure, such as the need for intraperitoneal access and dissection, dehiscence of primary intestinal anastomosis, bladder and rectal perforation, graft necrosis and scar fibrosis. There is the possibility of neovagina prolapse described in the long term.

### What is the criterion for success?

Although many studies report a desirable length of 6 cm, the criterion for success should be based on the individual report of a functional vagina, that is, one that allows comfortable sexual activity. It is important not to promise a minimum vaginal size and to make it clear to the patient that vaginal length is not related to sexual satisfaction.

### How should gynecological care be guided in the postoperative period?

After McIndoe vaginoplasty surgery, the patient remains on complete bed rest for approximately five days to avoid any movement of the vaginal mold. After this period, the mold is removed, the neovagina is assessed and the mold is reinserted. Guidance on the care required for insertion, removal and washing of the mold is provided. Postoperative follow-up visits are recommended one week after surgery, and monthly onwards.

### How to guide gynecological follow-up?

Both post-dilation and post-surgical follow-up, we recommend regular gynecological follow-up. During the assessment, we check for the presence of discharge, bleeding, pelvic pain or dyspareunia.

Vaginal diameter and length should always be observed so that vaginal stenosis can be diagnosed early and vaginal dilation can be reoriented and adjusted in the routine.

A speculum examination is also necessary, especially if there are any associated symptoms, while a cytological examination is not regularly recommended due to the absence of a cervix. Sexually transmitted infections should be avoided with the use of condoms, and immunization against HPV should also be recommended. The association with any other clinical conditions should also guide specific monitoring.

## Final considerations

The investigation of primary amenorrhea should include MRKH as one of the main diagnoses, and hormonal, genetic and imaging studies are necessary. Diagnostic laparoscopy is rarely necessary, since MRI provides sufficient information when evaluated by experienced professionals. Information about the treatment, sexual relationship and reproductive future should be transmitted clearly to the patient and, if necessary, to family members. Individual and collective psychological support should be suggested. The first-line treatment is vaginal dilation, which should be suggested from the end of adolescence and the beginning of adulthood, and only started when the patient is confident and mature enough to adhere to the procedure. Surgeries to create neovaginas should be reserved for the rare cases in which vaginal dilation has not been successful or for those who have jointly decided on the procedure with the multidisciplinary team.
